# Effect of Cu Alloying on Mechanical Properties of Medium-C Steel after Long-Time Tempering at 500 °C

**DOI:** 10.3390/ma16062390

**Published:** 2023-03-16

**Authors:** Pavel Salvetr, Aleksandr Gokhman, Milan Svoboda, Črtomir Donik, Ivana Podstranská, Jakub Kotous, Zbyšek Nový

**Affiliations:** 1 COMTES FHT a.s., Prumyslova 995, 334 41 Dobrany, Czech Republic; 2 Department of Physics, South Ukrainian National Pedagogical University (SUNPU), Staroprotfrankivska 26, 65020 Odessa, Ukraine; 3 Institute of Physics of Materials ASCR, Žižkova 22, 616 00 Brno, Czech Republic; 4 Institute of Metals and Technology (IMT), Lepi pot 11, 1000 Ljubljana, Slovenia

**Keywords:** long-time tempering, strengthening, Cu precipitate, medium-C steel, mechanical properties

## Abstract

This research studies the influence of the copper alloying of medium-carbon steel on mechanical properties after quenching and tempering at 500 °C. The microstructure was characterised using SEM, EBSD, TEM, and XRD analysis. The mechanical properties were comprehensively investigated using hardness measurements, tensile and Charpy impact tests and solid solution, grain boundary, dislocation, and precipitation strengthening contributions were estimated. Higher yield strength for Cu-alloyed steel was confirmed at about 35–73 MPa. The precipitation strengthening contribution from Cu precipitates in the range of 11–49 MPa was calculated. The interaction between Cu precipitates and dislocations retards the decrease in dislocation density. Similar values of effective grain size of martensite crystals were measured for Cu-alloyed and Cu-free steel as well. Copper alloyed steel exhibited significantly deteriorated impact toughness, total plastic elongation, and reduction of area. The size of Cu precipitates ranged from 8.3 nm after tempering at 500 °C for 6 h to 13.9 nm after tempering for 48 h.

## 1. Introduction

Low-alloyed medium-carbon steels are widely used as structural parts and components that require high strength, toughness, and good fatigue properties. The desired mechanical properties are usually achieved through heat treatment consisting of quenching and tempering or partitioning. The transition carbides are formed, retained austenite is decomposed, and cementite precipitates during tempering and partitioning [1,2,3]. The strength depends on the following strengthening contributions—solid solution, grain boundary, dislocation, and precipitation. The solid solution strengthening is provided by the chemical composition of the steel. Grain boundaries generally influence the strength according to the Hall-Petch relation. However, grain boundaries without impurities and segregation are important for good mechanical properties. Thermomechanical treatment before quenching improves mechanical properties [4,5]. Dislocations have an important effect on mechanical properties and the dislocation density value decreases during tempering. The strengthening contribution of precipitates includes transition carbide, cementite, and other precipitates. In particular, the combination of precipitated transition carbides and high dislocation density after low-temperature tempering provides very high strength [6]. In particular, the alloying of steels with copper is seen as an effective way to further increase the strength of steels. Especially in near-pure iron and low-carbon steels, high yield strength increases of up to 300 MPa can be achieved [7,8,9,10,11,12], but the strengthening effect of copper is also observed in medium-carbon steels, precipitation-hardened martensitic steel, and other steels [13,14,15,16,17,18]. The solubility of Cu in ferrite is very low, and Cu precipitates out from a solid solution when the Cu content is higher than 0.5 wt.% [9,16]. The copper usually precipitates between 400 and 650 °C in several steps: formation of Cu-rich clusters, body-centred cubic (bcc) Cu, 9R (orthorhombic lattice), and finally, stable face-centred cubic (fcc) Cu precipitates [9,14,19]. The influence of martensite deformation on Cu precipitation strengthening during tempering was investigated in [8]. Non-rolled martensite exhibited a predictable behaviour—the precipitation strengthening intensified at higher tempering temperatures and with longer tempering times. The precipitation strengthening did not exhibit dependence on tempering time and temperature in cold-rolled martensite. A higher increase in the yield strength was observed for non-rolled material (200 MPa) compared to 120 MPa in cold-rolled material [8]. The solid solution strengthening by Cu occurs when Cu is solute in a ferrite matrix. However, this strengthening mechanism only occurs in the case of low dislocation densities [20]. Therefore, no strengthening effect of copper was observed in previous work [13]. On the contrary, a deterioration of toughness and plastic properties was found in Cu-containing steels [13,21].

This study aims to compare mechanical properties using the tensile test, hardness measurement, and Charpy impact test of Cu-free and Cu-containing medium-C 1.7102 steels. Because this study requires the occurrence of copper precipitates in a microstructure, long-time tempering at 500 °C for tempering times up to 48 h was carried out. The presence of Cu precipitates in the microstructure was observed by transmission electron microscopy (TEM). X-ray diffraction (XRD) was used to determine the dislocation density, and electron back-scattered diffraction provided information about the effective grain size.

## 2. Materials and Methods

### 2.1. Materials and Heat Treatment

**Figure 1:**
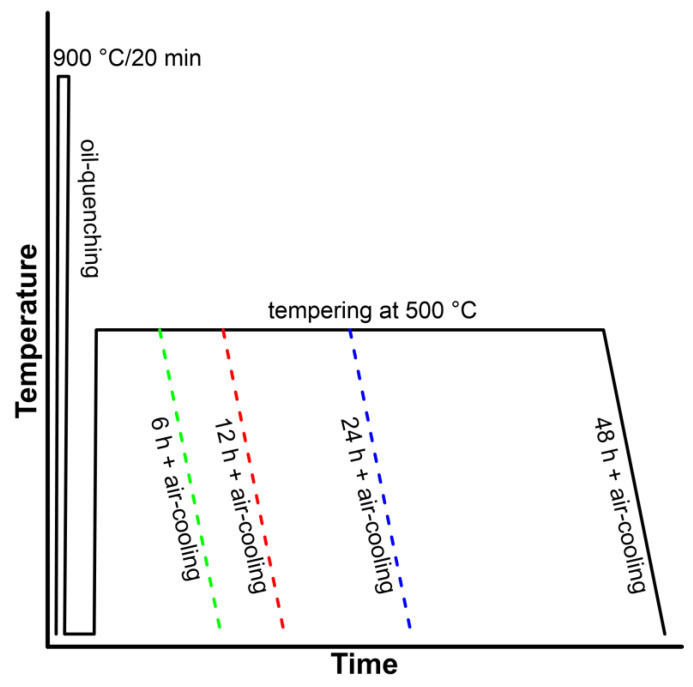
Schematic illustration of the heat treatment.

Two medium-C steels with and without Cu were studied. The chemical compositions of both materials are given in Table 1. Experimental steels were prepared by vacuum induction melting in the COMTES FHT company and casted into 45 kg ingots; ingots were heated to 1050 °C and subsequently hot rolled to 14 mm thick plates and air-cooled. The plates were normalizing annealed at 850 °C for 40 min. Cylindrical samples 13 mm in diameter and 120 mm in length were machined and subjected to various treatment regimes. First, all samples were heated to the same austenitization temperature of 1173 K (900 °C) for 20 min and oil-quenched. Then, both materials were tempered at 500 °C for 6 h, 12 h, 24 h and 48 h, followed by air cooling, as shown in the heat treatment diagram (Figure 1).

### 2.2. Mechanical Properties

Mechanical properties were tested by tensile and Charpy impact tests. Round tensile samples of 50 mm in gauge length and 8 mm in diameter were tested at a rate of 0.75 mm/min on a Zwick Z250 testing machine with a 250 kN capacity according to ČSN EN ISO 6892-1. Tensile characteristics were evaluated (e.g., Ultimate tensile strength—R_m_; Yield strength—R_p0.2_; Young modulus—E; uniform plastic elongation—A_g_; total plastic elongation after a fracture—A_5_; and reduction of area—Z). Hardness measurement was carried out on a Vickers hardness tester (Struers Durascan 50, Copenhagen, Denmark) using a diamond indenter under the load of 10 kg for 10 s (ČSN EN ISO 6507-1) 10 times for each sample. The Charpy V-notch impact tests were conducted at ambient temperature using a WPM PSd 300 J Charpy pendulum according to ČSN EN ISO 148-1. Charpy V notch specimens were prepared with dimensions 55 mm × 10 mm × 5 mm and a 2 mm deep V notch. Three tests were conducted for each condition, and the average value was calculated.

### 2.3. Microstructure Characterization

The samples for microstructure observations were polished, the final step using colloidal silica with a particle size of 0.05 μm. The microstructure was revealed by etching in Nital reagent (98 mL of ethanol + 2 mL nitric acid). Next, the microstructure was observed by scanning electron microscope (SEM) JEOL IT 500 HR (JEOL, Tokyo, Japan). Electron Back-Scatter Diffraction (EBSD, Hikari Super camera, EDAX LLC, Mahwah, NJ, USA) was used to determine crystallographic features such as grain boundaries, grain size, and dislocation density distribution. EBSD analysis was performed with a scanning step of 0.05 μm on an area of 40 × 40 μm.

A transmission electron microscope (TEM) JEOL JEM-2100F (JEOL, Tokyo, Japan) and Talos F200i (Thermo Fisher Scientific, Brno, Czech Republic) equipped with an energy dispersive X-ray spectrometer (EDS) were used for a more detailed analysis of the microstructure and identification of copper-based and carbide particles on EDS maps of chemical composition. Thin foils were prepared using a Struers Tenupol 5 twin-jet electropolishing system (Struers ApS, Ballerup, Denmark) in a solution of perchloric acid (5 mL) and methanol (95 mL) at −20 °C. Carbon replicas were prepared to determine the chemical composition of the cementite.

The phase composition and dislocation density and residual stress were determined by a BRUKER D8 Discover diffractometer (Bruker AXS GmbH, Karlsruhe, Germany) with a Cu anode (λ = 0.15406 nm, 40 kV, 40 mA) in Bragg-Brentano geometry. The X-ray diffraction (XRD) lines were recorded from 30 to 120° in 2θ mode with a step size of 0.025. The dislocation density (*ρ*) was estimated using the modified Williamson-Hall method [22,23] from the full width at the half maximum (FWHM) and the diffraction angle of ferrite peaks α(110), α(002), α(121), and α(022). The polished samples were etched in hydrochloric acid for 120 min to remove the surface layer and minimize the influence of defects originating from the polishing process.

## 3. Results

### 3.1. Mechanical Properties

**Figure 2:**
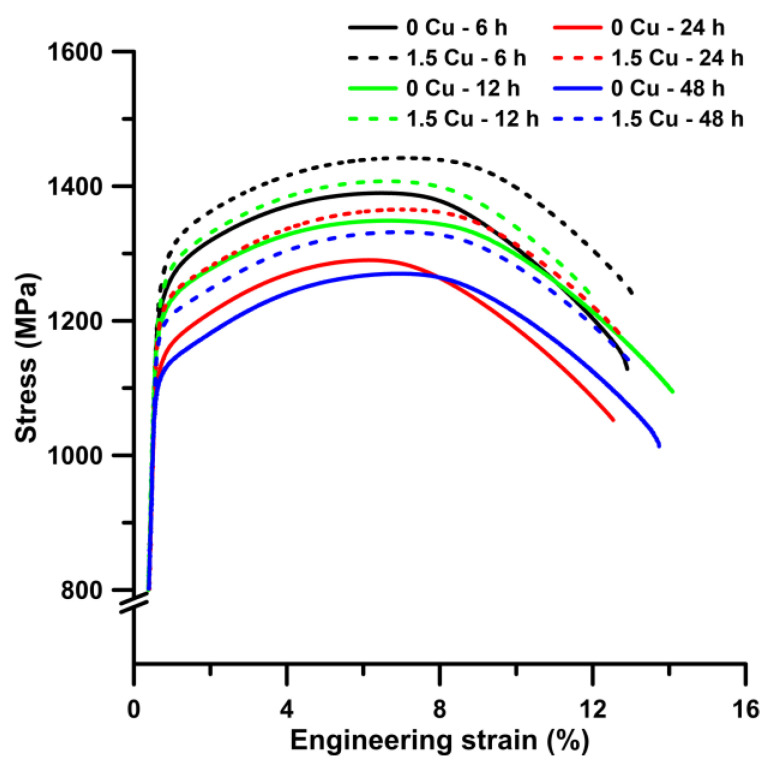
Tensile engineering stress—strain diagram for steels 0 Cu and 1.5 Cu tempered at 500 °C.

The results of the tensile, Charpy impact, and hardness tests are summarized in Table 2, and the engineering stress-strain curves obtained at room temperature are shown in Figure 2. The yield and ultimate tensile strength of both steels decreased with the longer tempering times. The differences between the yield strength of steel 1 Cu and 0 Cu increase continually with a longer time of tempering up to 24 h. After that, the difference levelled out at approx. 70 MPa. The values of total plastic elongation remained unchanged between 11% and 13% during tempering. The reduction of area of the 0 Cu samples reached high values of about (40%) for each tempering time, and its values were significantly higher than the 1.5 Cu sample. In the 1.5 Cu sample, the reduction of area was about 28% for 6 h and 12 h long tempering and then increased to 34% and 36% for 24 h and 48 h long tempering. Hardness measurements confirmed the results of ultimate tensile strength. The hardness of 1 Cu steel is about 34 HV10 higher on average, and the hardness values of both materials decreased gradually with the longer tempering time.

**Figure 3:**
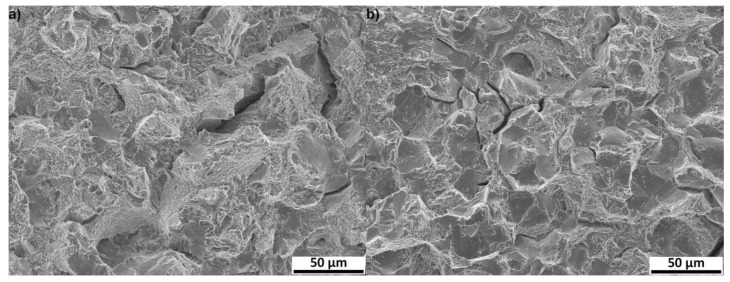
Charpy impact test fracture surfaces of samples after tempering for 24 h: 0 Cu steel (**a**) and 1.5 Cu steel (**b**) observed by SEM in a secondary electrons mode (SE).

The impact toughness determined using the Charpy impact test revealed almost unchanging impact toughness of steel 0 Cu during tempering at about 27 J/cm^2^. The impact toughness of the 1.5 Cu steel was lower than in 0 Cu, and its value varied between 17 J/cm^2^ and 21 J/cm^2^. Fracture surfaces of both steels after tempering for 24 h were examined using SEM (Figure 3). There are typical features for a mixed mode of intergranular and transgranular fracture. The intergranular region had a faceted appearance, whereas transgranular regions showed dimple morphology. The reason for the higher value of KCV in the 0 Cu sample than in the 1.5 Cu sample were the larger areas of ductile fracture with dimples. Deep cracks were also observed on the fracture surfaces of both samples.

### 3.2. Microstructure Characterization

**Figure 4:**
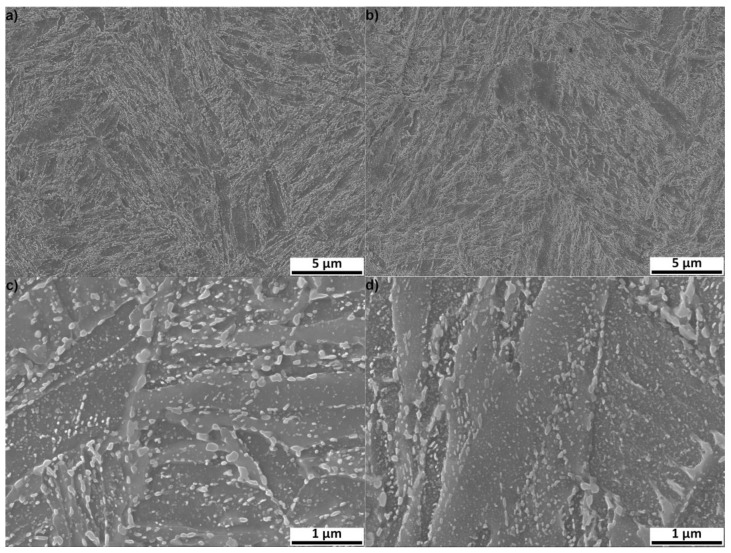
SEM-SE micrographs of samples 0 Cu tempered for 6 h (**a**); 1.5 Cu tempered for 6 h (**b**); 0 Cu sample tempered for 24 h (**c**); and 1.5 Cu sample tempered for 24 h (**d**).

The microstructures of both 0 Cu and 1.5 Cu materials consisted of a martensitic matrix and cementite (see Figure 4a,b) of 0 Cu and 1.5 Cu samples tempered at 500 °C for 6 h. The size of martensitic lath is mostly within the range for a length of 1–3 μm and for a width of 0.3–0.6 μm. However, martensitic laths with a length of about 10 μm and width of 4 μm were also occasionally found. Larger particles were formed during tempering at the boundaries of martensitic crystals, whereas finer cementite particles were formed within martensitic crystals. The microstructures of both steels look similar, but it seems that the interior of the martensitic lath contains more very fine particles, as is presented in the more detailed SEM micrographs of the 0 Cu and 1.5 Cu samples tempered at 500 °C for 24 h in Figure 4c,d.

**Figure 5:**
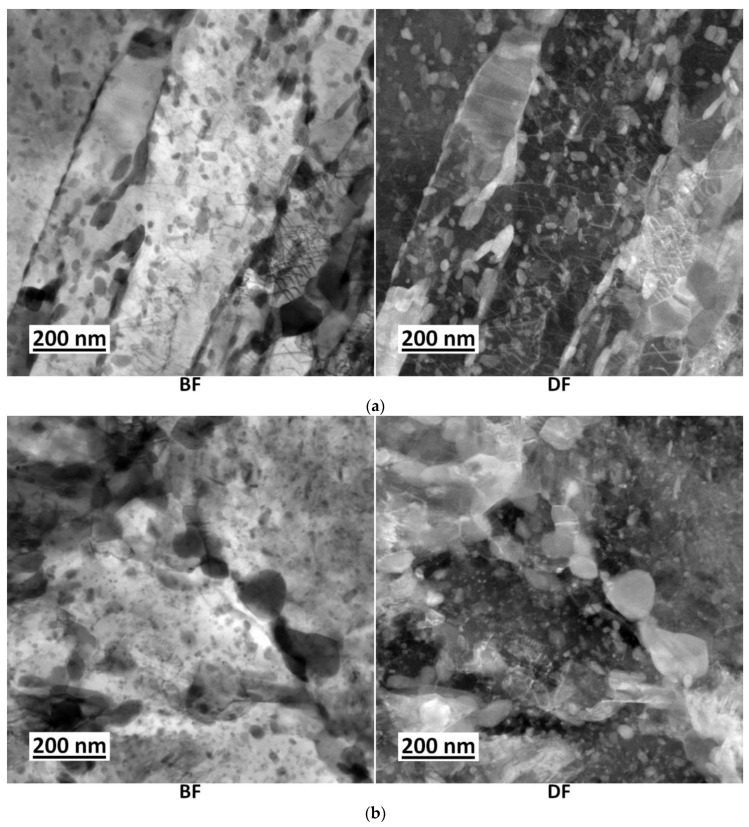

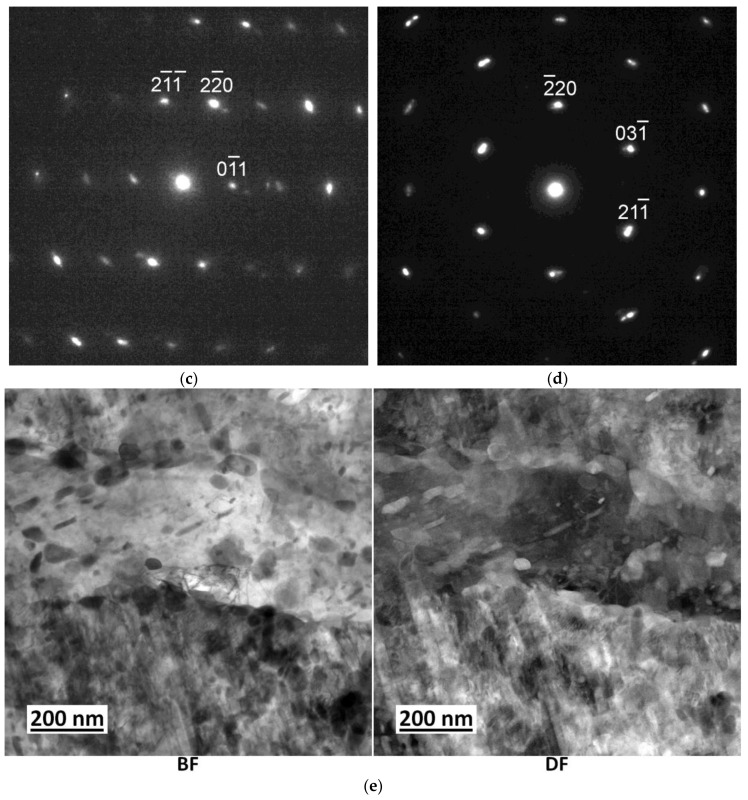

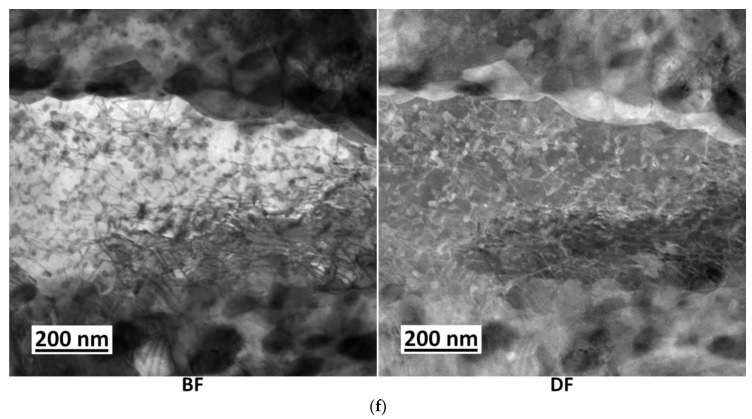
Cementite particles at PAG and martensite packet boundaries in 0 Cu (**a**) and 1.5 Cu (**b**) steel tempered for 24 h and corresponding SAED results of cementite in 0 Cu (**c**) with zone axis [111] and 1.5 Cu (**d**) steel with zone axis [113]. Comparison of density of fine precipitates in microstructure of 0 Cu (**e**) and 1.5 Cu (**f**) steels.

TEM was used for more detailed observation and analysis of the particles. The precipitates were compared in both materials after tempering at 500 °C for 24 h. In both materials, the large precipitates with a size of 50–200 nm occurred at the PAGBs and boundaries of martensite packets (Figure 5a,b). Cementite particles were identified using selected area electron diffraction (SAED), as shown in Figure 5c,d.

**Figure 6:**
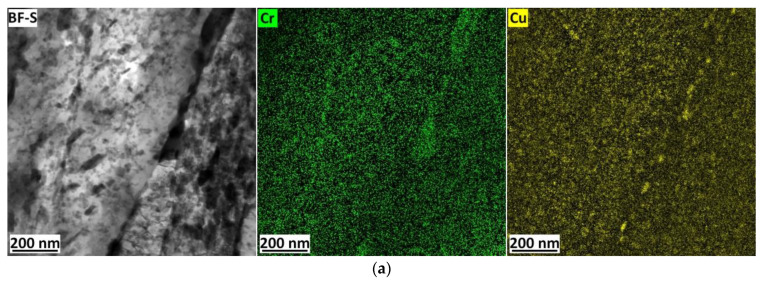

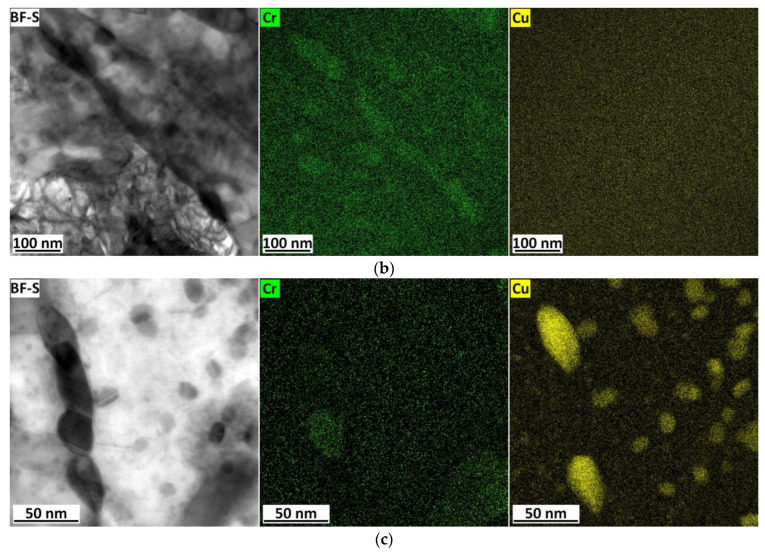
Precipitates in 1.5 Cu and 0 Cu samples tempered at 500 °C for 24 h (green coloured Cr map represents cementite particles, and the yellow colour represents EDS maps of Cu precipitates): Cu precipitates precipitated at martensitic needles boundary in the sample 1.0 Cu (**a**); martensitic needle boundary occupied by cementite in sample 0 Cu (**b**); and Cu precipitates formed at the edge of cementite inside the martensitic needle in the microstructure of 1.5 Cu tempered at 500 °C for 48 h (**c**).

A lot of fine precipitates with a size of tens of nanometres arose within the martensitic needles in both steels (Figure 5e,f). As proven by EDS analyses, these fine precipitates were cementite in both materials, and in the 1.5 Cu, there were additionally Cu precipitates with a size of about 10 nm. Thus, the density of fine precipitates seems to be higher in the 1.5 Cu sample. The resolution of carbides (cementite) and Cu-precipitates using EDS maps is reliable because the carbides are enriched in chromium and manganese. Cementite particles contained, on average, about 7.8 wt.% of Cr and 5.5 wt.% of Mn (balance Fe), according to the EDS analyses of the carbon replicas. An example of the occurrence of Cu particles at the boundaries of martensitic needles is shown in Figure 6a. An EDS map for Cr identifies the cementite precipitates. For comparison, the TEM micrograph in Figure 6b represents a similar EDS map also covering a boundary of a martensitic needle on the 0 Cu sample. Figure 6c demonstrates Cu precipitation occurring at the edge of cementite.

**Figure 7:**
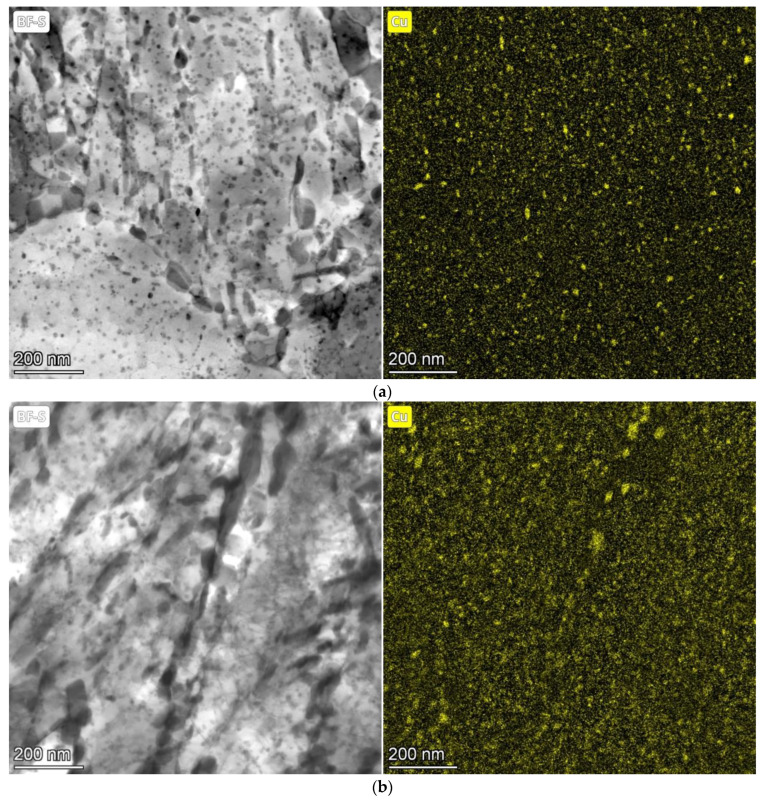

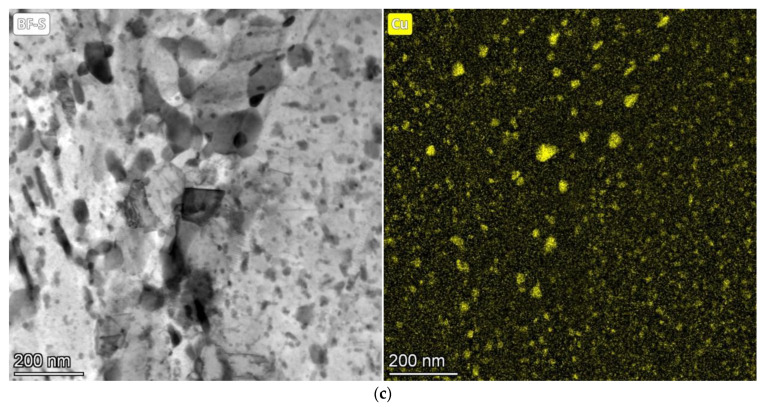
Evolution of Cu precipitates in 1.5 Cu steel during tempering at 500 °C for 12 h (**a**); 24 h (**b**); and 48 h (**c**).

The evolution of Cu precipitates was evaluated from the EDS map of chemical composition. Based on the EDS maps (Figure 7), the growth of Cu precipitates was summarized in Table 3 during tempering for 12 h, 24 h and 48 h, and the decrease in density of Cu precipitates was found. Some copper precipitates appear to be increasing at the expense of smaller precipitates. Cu precipitates occurred both within the martensite needles and at their boundaries (see Figure 6). At the boundaries of the martensitic needles, precipitates were preferentially at the edges of the cementite particles, where they grew to larger sizes than inside the martensitic needles. The Cu precipitates formed inside martensitic needles are shown in Figure 6.

As seen in Table 3, the growth of Cu precipitates occurred mainly within 24 h of tempering at 500 °C inside the martensitic needles. However, Cu precipitates at preferential localities (grain boundaries and edges of cementite particles) continued in growth.

**Figure 8:**
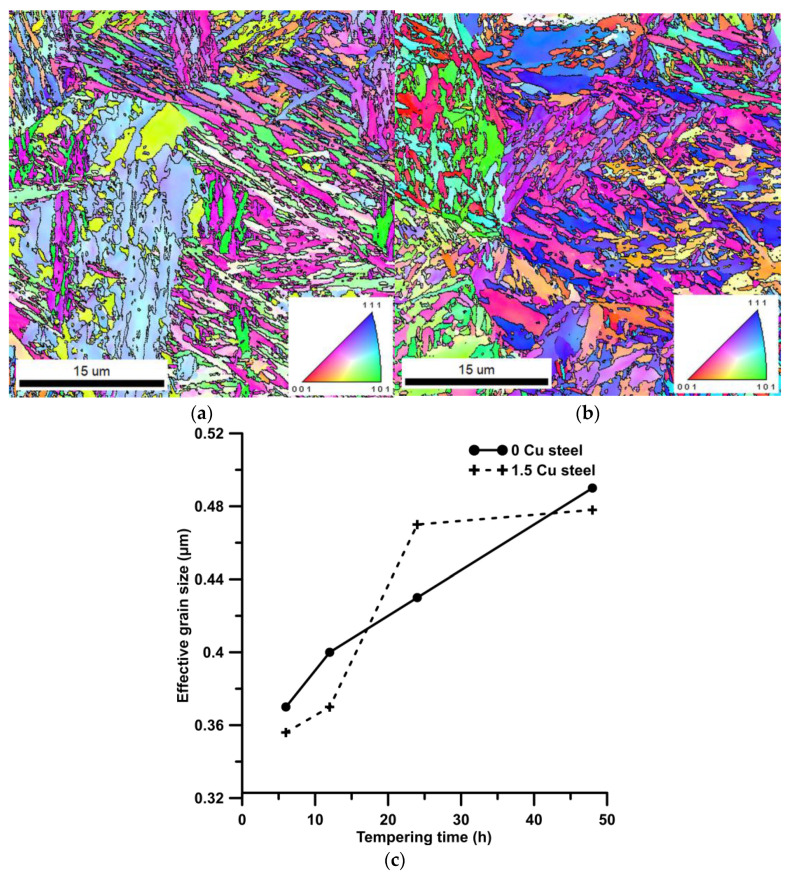
Grain orientation IPF EBSD maps of 0 Cu (**a**) and 1.5 Cu (**b**) steel tempered at 500 °C for 24 h. Black lines show high-angle grain boundaries with misorientation above 15°. Evolution of grain size during tempering (**c**).

EBSD analysis provided an estimation of effective grain size (EGS). Similar to the previous study [24], the high-angle grain boundaries (HAGBs) with misorientation above 15° were used to determine EGS. The inverse pole figure maps (IPF) of samples 0 Cu and 1.5 Cu tempered for 24 h are shown in Figure 8. HAGBs are marked by black lines. Values of EGS are presented in Figure 8. EGS increased slightly with a longer tempering time in both steels. Any effect of Cu addition or Cu precipitates on EGS was proven because of very small differences in the EGS of 0 Cu and 1.5 Cu samples.

**Figure 9:**
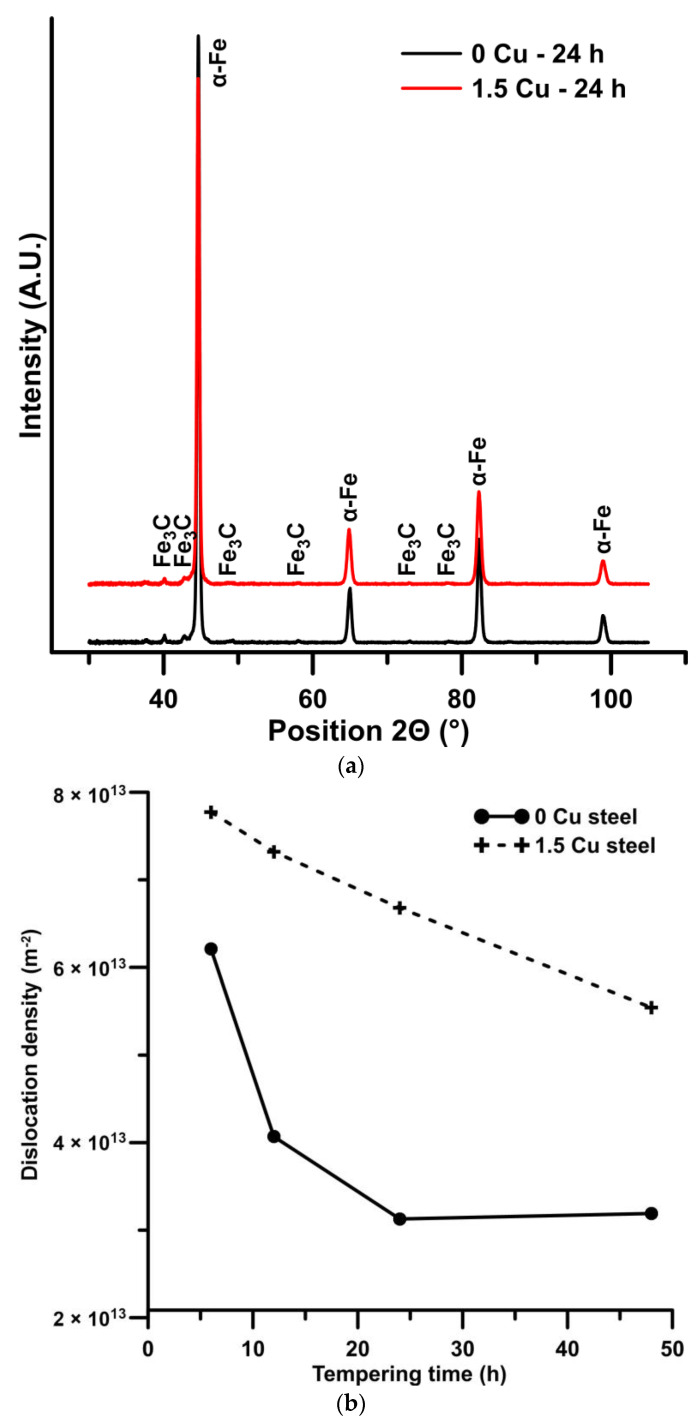
XRD patterns of 0 Cu and 1.5 Cu samples after tempering at 500 °C for 24 h (**a**) and dislocation density evolution during tempering at 500 °C (**b**).

Figure 9a shows the XRD patterns of both materials after tempering at 500 °C for 24 h. The phase composition consisted of ferrite, and a small amount of cementite was found by XRD analysis. No diffraction peak of retained austenite was detected. The dislocation densities were calculated according to the XRD results. Higher values of dislocation densities were calculated in the 1.5 Cu sample compared to the 0 Cu sample (see Figure 9b). Generally, dislocation densities decreased with longer tempering time in both samples. However, the decrease in the dislocation density of the 1.5 Cu sample occurred gradually, whereas the dislocation density dropped sharply up to the tempering time of 24 h in the 0 Cu sample, and after that, the value of dislocation density remained similar.

## 4. Discussion

The increase in strength and hardness via Cu addition in steel has been observed mainly in low-carbon steels [9,12,16,17]. However, several works also studied the strengthening by Cu in medium-carbon steel [14,25]. Any increase in strength was determined using tensile tests in the quenched and tempered steel 1.7102 up to the tempering temperature of 400 °C. However, the mechanical properties of this steel with copper addition were lower compared to the same Cu-free steel. The strengthening due to copper addition was first measured after tempering at 500 °C [13]. Another study compared the hardness of Cu-alloyed and Cu-free medium-carbon-bearing steel after tempering at 450 °C, and the decrease in hardness was gentler in Cu-alloyed steel than in Cu-free steel [14,25]. Thus, this work described the effect on the mechanical properties of medium-C steel in detail.

According to previous studies [9,24,26], the yield strength of steel can be expressed as Equation (1), and it is dependent on the following factors: lattice friction stress (*σ*_0_), solid solution strengthening (*σ_ss_*), grain boundary strengthening (*σ_g_*), dislocation strengthening (*σ_d_*), and precipitation strengthening (*σ_p_*).

(1) $$\sigma_{YS}=\sigma_{0}+\Delta \sigma_{SS}+\Delta \sigma_{g}+\Delta \sigma_{d}+\Delta \sigma_{p}$$

The lattice friction stress of ferrite is estimated to be approximately 85 MPa. The solid solution strengthening contribution is based on the chemical composition of the steel due to differences in the atom size between the solute and solvent (iron atoms in steel) atoms, the strain field that interferes with the dislocations as they move through the lattice causing plastic flow, and differences in the shear modulus [27]. The empirical values of solid solution strengthening factors for individual alloying elements per 1 wt.% (*A*_i_) for the estimation of the degree of the solid solution strengthening are as follows: 0 MPa (Cr) [27], 83 MPa (Si) [24], 33 MPa (Ni) [27], 32 MPa (Mn) [24], and 39 MPa (Cu) [24]. The solid solution strengthening is given by Equation (2), where *A_i_* is a factor for strengthening resulting from 1.0 wt.% addition of elements *i*, and wt.% is the content of the element *i* in steel in wt.%. The chemical composition of ferrite does not correspond to the chemical composition of steel due to carbide and copper particles precipitation. Therefore, the calculated chemical composition of ferrite using JMatPro software (in Table 4) under equilibrium conditions at 1100 °C was used for estimating the solid solution strengthening contribution.

(2) $${\Delta \sigma }_{SS}=\sum A_{i}\times wt.\%$$

The solid solution strengthening contribution can theoretically reach a value of 126 MPa for 0 Cu steel and a value of 186 MPa for 1.5 Cu steel if all alloying elements are solute in a solid solution of ferrite. However, both steels were tempered at 500 °C for many hours, thus, Cr precipitated in cementite particles and Cu precipitates were formed. Therefore, we assumed that the difference in solid solution strengthening is approximately 152 MPa for both steels. Moreover, Cu contribution to solid strengthening was not found after low-temperature tempering [13] because the solid strengthening by copper deteriorates and disappears when dislocation density is high [20].

The Hall–Petch relationship (Equation (3)) provides an estimation of strengthening contribution from grain boundaries. In Equation (3), the *k*_y_ (0.2 MPa·m^−1/2^) [24,28,29] is the Hall–Petch slope representing the potency of grain boundary strengthening, and *d* is grain size. In this work, *d* was determined using EBSD. HAGBs with a misorientation angle above 15° are considered as arrest dislocation motions and were used in the Hall–Petch relationship.

(3) $$\Delta \sigma_{g}=k_{y}d^{-\frac{1}{2}}$$

The dislocation strengthening to yield strength was calculated according to the Taylor equation (Equation (4)), where *α* = 0.25 (dislocation obstacle efficiency coefficient) [24,30], *M* = 3 (Taylor factor) [24,30], *G* = 76 GPa (shear modulus) [24,31], *b* = 0.248 nm (Burgers vector) [24], and *ρ* = dislocation density calculated from the XRD results (Figure 9) according to the Williamson–Hall method.

(4) $$\Delta \sigma_{d}=\alpha MGb\sqrt{\rho }$$

Precipitation strengthening contribution can be estimated by using the Ashby–Orowan equation (Equation (5)), where *V_f_* is the volume fraction, and *X* represents the diameter of the particle in mm, taken to be an equivalent spherical diameter of precipitates, similar to the work [32].

(5) $$\Delta \sigma_{p}=\left(\frac{0.538Gb\sqrt{V_{f}}}{X}\right)ln\left(\frac{X}{2b}\right)$$

The contribution of precipitation strengthening to yield strength consists of contributions of Cu precipitates, cementite, and possibly other carbides such as Cr_23_C_6_. Therefore, all other contributions to the yield strength such as solid solution strengthening, dislocation strengthening, lattice friction strengthening, and grain boundary strengthening were determined, and yield strength values were also measured by tensile testing. The precipitation strengthening contribution can be calculated by subtracting individual strengthening contributions (*σ*_0_, *σ_SS_*, *σ_d_*, *σ_b_*) from the yield strength for each sample (Equation (6)). We assumed that the precipitation strengthening contribution from carbides is the same because of similar chemical compositions and applied identical heat treatment processing of both 0 Cu and 1.5 Cu steel. Then, the precipitation strengthening contribution from Cu precipitates (Δ*σ_p_*_-*Cu*_) is equal to the difference of the precipitation strengthening contributions of 1.5 Cu steel and 0 Cu steel for each tempering time, according to Equation (7).

(6) $$\Delta \sigma_{p}=R_{p0.2}-\sigma_{0}-\Delta \sigma_{SS}-\Delta \sigma_{b}-\Delta \sigma_{d}$$

(7) $$\Delta \sigma_{p-Cu}=\Delta \sigma_{p-1.5\;Cu\;sample}-\Delta \sigma_{p-0\;Cu\;sample}$$

The values of individual strengthening contributions are summarized in Table 5. The values of the strengthening contributions such Δ*σ_p_*, Δ*σ_p-Cu_* and differences between yield strength of 1.5 Cu and 0 Cu samples (Δ*R_p_*_0.2_) were also calculated. All 1.5 Cu samples reached higher yield strengths than the 0 Cu samples. The difference between the yield strengths of 1.5 Cu and 0 Cu samples increased with longer tempering times, but the main increases between these yield strengths appeared between the tempering times of 12 h and 24 h. This tempering period also includes an increase in yield strength attributed to precipitation strengthening from Cu precipitates (Δ*σ_p-Cu_*). An increase in the size of the precipitated Cu was observed by TEM during these tempering times. Cu precipitates also interact with dislocations, as shown in the TEM micrograph (Figure 5f), and probably cause a slowing down of dislocation density decrease, as indicated by XRD results in Figure 9.

The findings described in this work are in good agreement with the previous investigation focused on the same grade of steel. Jung et al. [14] followed the strengthening of Cu precipitates by hardness measurement after quenching and during tempering at the temperature of 450 °C. The as-quenched 0 Cu and 1.5 Cu steels exhibited similar values of hardness, and the difference in hardness between 0 Cu and 1.5 Cu steels gradually increased with further tempering time up to approx. 27 h. Then, the increase in hardness was stopped. Jung et al. [14] also studied the atomic structure of Cu precipitates during tempering and considered the Cu precipitates with a particle diameter of approximately 5 nm or smaller as bcc precipitates coherently with the martensite matrix. Larger precipitates with diameters ranging between 5–15 mm were described as orthorhombic 9R Cu precipitates. Within the precipitates larger than approximately 15 nm, the crystal structure of Cu precipitates is thought to be 3R or fcc. B. Kim et al. [24] also reported a strengthening contribution model for medium-C steel with a similar chemical composition. A higher yield strength of approximately 300 MPa was reported due to the lower tempering temperature of 450 °C and the shorter tempering time of 30 min. Thus, only a rough comparison of strengthening contributions can be made. From the dislocation strengthening point of view, our study reported a significantly lower strengthening contribution from dislocations resulting from higher tempering temperatures and longer tempering times. The precipitation strengthening contribution of 587 MPa was reported by B. Kim et al. [24]. It means that our estimate of Δσ*_p_* is slightly lower for the 0 Cu sample and slightly higher for 1.5 Cu steel compared to [24] for the tempering time of 6 h.

From the precipitation strengthening contributions, it is possible to estimate the volume fraction of Cu precipitates in the microstructure based on Equation (5) and the mean Cu particle sizes measured during TEM analysis shown in Table 3. The calculated volume fractions of Cu precipitates are compared to the volume fraction estimated by JMatPro software for equilibrium conditions at 500 °C in Table 6. The values of the volume fraction of Cu precipitates were similar for equilibrium conditions according to the JMatPro Software and for the tempering time of 24 h.

The results in Table 6 can be explained as follows. The tempering time of 12 h is not a sufficiently long tempering time for the complete precipitation of copper from the solid solution of ferrite. The tempering time of 24 h is sufficient for Cu precipitation and at the same time, the size and homogenous distribution of precipitates in the microstructure are important for the strengthening effect. The Cu precipitates grow only slightly after tempering for 48 h compared to the tempering time of 24 h, but it can be concluded from TEM micrographs and EDS maps that the density of the precipitates decreases, and only Cu precipitates continue to grow in areas of preferential occurrence—see TEM micrographs and EDS maps in Figure 7. Therefore, the potential of Cu precipitates for strengthening is not fully utilized. 

When comparing the effect of copper on strengthening and yield strength between medium-carbon steels and low-carbon steels, there is a substantial difference. Although in this work an increase in yield strength of maximum 73 MPa was measured, in the case of lower carbon or near-pure iron, an increase in yield strength of 220 MPa and 300 MPa, respectively, was found [7,9].

## 5. Conclusions

1. Copper alloying of medium-C steel increases the yield strength and ultimate tensile strength of the steel after tempering at 500 °C. The negative effect of Cu in steel is manifested by a deterioration in ductility and a reduction of area. The impact test also demonstrated a significant decrease in impact toughness.
2. Determination of the precipitation strengthening contribution is difficult due to the mixture of precipitates formed in the microstructure, including Cu precipitates but also cementite and other carbides. Therefore, the precipitation strengthening contribution was determined by subtracting the individual strengthening contributions such as lattice friction, solid solution, grain boundary, and dislocation strengthening from the value of yield strength evaluated by the tensile test. The precipitation strengthening contribution of Cu precipitates was determined as the difference in precipitation strengthening of the 1.5 Cu steel and Cu-free steel. Strengthening of approximately 15 MPa represents the precipitation strengthening from Cu-precipitates for tempering times of 6 and 12 h. A precipitation strengthening from Cu precipitates of 45 MPa was determined for tempering times of 24 h and 48 h.
3. Overall, higher yield strengths of 35 MPa, 54 MPa, 70 MPa, and 73 MPa were measured for 1.5 Cu steel for tempering times of 6 h, 12 h, 24 h and 48 h. The increase in yield strength for steels with medium-C contents is not nearly as effective as for steels with low contents of carbon and other alloying elements or almost pure iron, where a yield strength of 200–300 MPa higher was found for Cu-alloyed steels compared to Cu-free steels.
4. Cu precipitates grow the most during tempering up to 24 h and reach an average size of 13.4 nm. During longer tempering times, only Cu precipitates grow at preferential locations of occurrence, and the density of copper precipitates decreases.
5. Copper precipitates retard the decrease in dislocation density; however, an effect on grain size was not found.
6. Martensite deformation could induce Cu precipitation strengthening at lower tempering temperatures and shorter tempering times. A long-time tempering at 500 °C accompanied by a drastic decrease in the strength of steels could be omitted from the heat treatment.

## Figures and Tables

**Table 1:** Chemical compositions of the investigated steels in wt.%, balance Fe.

Steel	C	Si	Mn	Cr	Ni	Cu	Fe
1.5 Cu	0.54	1.50	0.74	0.76	0.074	1.51	Bal.
0 Cu	0.55	1.51	0.71	0.79	0.108	0.04	

**Table 2:** Summary of mechanical properties—tensile yield strength (R_p0.2_), ultimate tensile strength (R_m_), total plastic elongation (A_5_), reduction of area (Z), and hardness and Charpy impact toughness (KCV).

Properties	Sample	Tempering Time (h)			
		6	12	24	48
R_p0.2_	0 Cu	1248 ± 25.0	1211 ± 9.3	1147 ± 3.4	1119 ± 5.6
	1.5 Cu	1283 ± 4.7	1255 ± 4.8	1217 ± 18.3	1192 ± 10.9
R_m_	0 Cu	1388 ± 10.8	1352 ± 9.7	1294 ± 5.7	1271 ± 7.6
	1.5 Cu	1441 ± 4.7	1407 ± 5.4	1364 ± 10.1	1329 ± 7.7
A_5_	0 Cu	13 ± 0.5	13 ± 0.8	13 ± 0.2	12 ± 0.1
	1.5 Cu	12 ± 1.2	11 ± 0.3	11 ± 0.3	12 ± 0.7
Z	0 Cu	39 ± 1.6	40 ± 2.1	43 ± 0.8	42 ± 0.7
	1.5 Cu	29 ± 4.8	28 ± 2.6	34 ± 1.6	36 ± 4.1
Hardness (HV 10)	0 Cu	416 ± 2.1	400 ± 3.9	396 ± 2.6	388 ± 2.4
	1.5 Cu	450 ± 1.8	438 ± 2.0	425 ± 2.4	418 ± 3.2
KCV (J/cm^2^)	0 Cu	29 ± 0.3	27 ± 0.9	28 ± 0.5	29 ± 0.4
	1.5 Cu	17 ± 0.7	19 ± 0.9	18 ± 1.1	19 ± 0.6

**Table 3:** The size evolution of Cu precipitates inside the martensitic needles after tempering at 500 °C for 12 h, 24 h and 48 h.

Sample	Cu precipitate Size (Diameter in nm)	Standard Deviation (in nm)
1.5 Cu—500 °C—12 h	8.3	2.5
1.5 Cu—500 °C—24 h	13.6	2.6
1.5 Cu—500 °C—48 h	13.9	3.6

**Table 4:** Equilibrium chemical composition of ferrite at 500 °C according to JMatPro 13 software.

Element	Cr	Si	Ni	Mn	Cu
Equilibrium chemical composition of ferrite (wt.%)	0.07	1.66	0.08	0.21	0.06 *

* 0.04 wt.% of Cu for 0 Cu steel.

**Table 5:** Strengthening contributions—lattice friction stress (*σ*_0_), solid solution strengthening (*σ_SS_*), grain boundary strengthening (*σ_g_*), dislocation strengthening (*σ_d_*), strengthening from precipitates (*σ_p_*), and strengthening only from Cu-precipitates (*σ_p-Cu_*).

Steel	Tempering Time (h)	Strengthening Contributions (MPa)					
		*σ*_0_	*σ_SS_*	*σ_g_*	*σ_d_*	*σ_p_*	*σ_p-Cu_*
0 Cu	6	85	152	329	111	571	-
	12	85	152	316	90	558	-
	24	85	152	305	81	524	-
	48	85	152	286	80	516	-
1.5 Cu	6	85	152	335	125	586	15
	12	85	152	329	121	568	11
	24	85	152	292	116	573	49
	48	85	152	289	105	560	44

**Table 6:** Estimated volume fraction of Cu precipitates and volume fraction of Cu precipitates according to JMatPro software under equilibrium conditions at 500 °C.

Tempering Time (h)	12	24	48
*V_f_* of Cu precipitates (%)	0.0031	1.37	1.15
Equilibrium *V_f_* of Cu precipitates	1.45		

## Data Availability

The data presented in this study are available on request from the corresponding author.
